# A proxy-year analysis shows reduced soil temperatures with climate warming in boreal forest

**DOI:** 10.1038/s41598-018-35213-w

**Published:** 2018-11-15

**Authors:** Md Abdul Halim, Sean C. Thomas

**Affiliations:** 1 University of Toronto, Faculty of Forestry, 33 Willcocks Street, Toronto, ON M5S 3B3 Canada; 2 Shahjalal University of Science and Technology, Dept. of Forestry and Environmental Science, Sylhet, 3114 Bangladesh

## Abstract

Scientists unequivocally agree that winter air temperature (T_A_) in northern high latitudes will increase sharply with anthropogenic climate change, and that such increases are already pervasive. However, contrasting hypotheses and results exist regarding the magnitude and even direction of changes in winter soil temperature (T_S_). Here we use field and satellite data to examine the ‘cold soil in a warm world’ hypothesis for the first time in the boreal forest using a proxy year approach. In a proxy warm year with a mean annual temperature similar to that predicted for ~2080, average winter T_S_ was reduced relative to the baseline year by 0.43 to 1.22 ^°^C in open to forested sites. Similarly, average minimum and maximum winter T_S_ declined, and the number of freeze-thaw events increased in the proxy warm year, corresponding to a reduction in the number of snow-covered days relative to the baseline year. Our findings indicate that early soil freezing as a result of delayed snowfall and reduced snow insulation from cold winter air are the main drivers of reduced winter active-layer T_S_ (at ~2-cm depth) under warming conditions in boreal forest, and we also show that these drivers interact strongly with forest stand structure.

## Introduction

Anthropogenic climate change is predicted to increase global average air temperature (T_A_) 1.4–5.8 ^°^C by 2100, with substantially higher increases in winter T_A_ in northern high latitudes^1^ and concomitant effects on the timing, form, and amount of precipitation^1,2^. In northern high latitude ecosystems (boreal forests and tundra) that occupy ~22% land area and store ~40% soil carbon globally^3^, soil temperature (T_S_) may respond differently than T_A_ due to the decoupling effects of snow cover^4,5^. Snow manipulation experiments have indicated large impacts of snow cover on T_S_ regimes and responses of soils and vegetation^2,6,7^. Changes in freeze-thaw events (FTEs) are of particular concern^2^, posing an “agent of surprise”^8^ in the functioning of northern ecosystems, with large potential effects on root mortality^9^, post-winter sapling survival^7^, soil nutrient losses^10^, soil microbial activities^11,12^, and the stability of stored carbon^13,14^.

T_S_ data, particularly under field conditions, are scarce compared to T_A_^4^, and climate-model-prediction scenarios have typically only been developed for T_A_^5^. Several snow manipulation studies^2,15,16^ have suggested that in a warmer world soils during winter months may be colder as a result of decreased and delayed snow insulation. In contrast, most simulation models^4,5,17^ have predicted a rise in T_S_ in warm climates, as a synergistic effect of increased T_A_ and reduced snowfall, though a few models^18,19^ do suggest that climate warming could reduce T_S_ under some conditions. T_S_ measurements in high latitude ecosystems have commonly been made at a depth of ≥10 cm, but the soil is most responsive and biologically active above 10 cm soil depth^10,20^. Contradictory model predictions indicate that T_S_ sensitivity to climate change is not well understood^18^, and a lack of data to test alternative models has been noted^10^.

**Figure 1:**
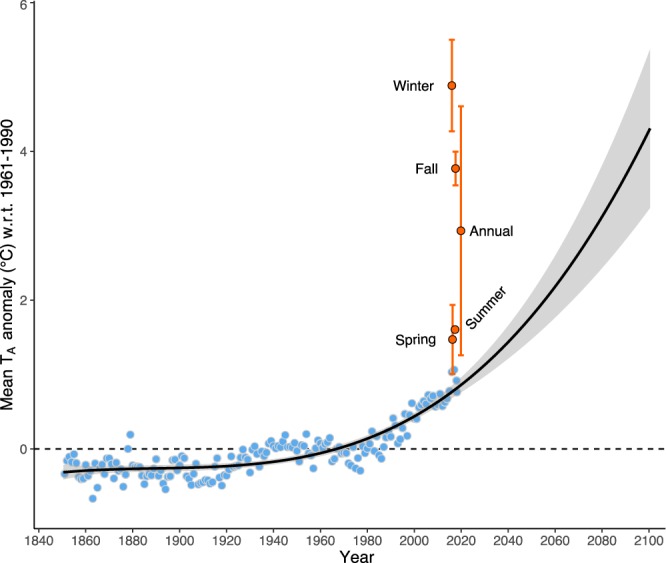
Local T_A_ anomalies compared against the HadCRUT4 northern hemisphere (NH) anomalies to establish proxy years. A polynomial regression curve (solid black line) (with standard errors in grey shading) is fitted to the annual HadCRUT4 data (baseline 1961–1990) (blue circles) to show a general anomaly trend for NH over the 21^st^ century. Y_W_ (December 2015–November 2016) local annual and seasonal anomalies (orange circles) (with standard deviations) are significantly higher (p < 0.01) than those for NH and Y_B_ (December 2013–November 2014). Y_B_ local anomalies are not significantly different than NH anomalies, thus are not presented here.

Here we provide a first test of the ‘cold soil in a warm world’^15^ hypothesis in the boreal forest using a proxy year approach, making use of recent climate variability to compare T_S_ patterns between a proxy baseline year (Y_B_) and a warm future year (Y_W_) (Fig. 1). We used soil and micrometeorological tower sensor data from study plots distributed in open, partially forested, and forested sites in a mixedwood boreal forest in northwestern Ontario, Canada. Snow cover durations were inferred from diurnal T_S_ patterns and confirmed using synthetic high-resolution imagery (fusing MODIS and Landsat 8 data).

## Results

**Figure 2:**
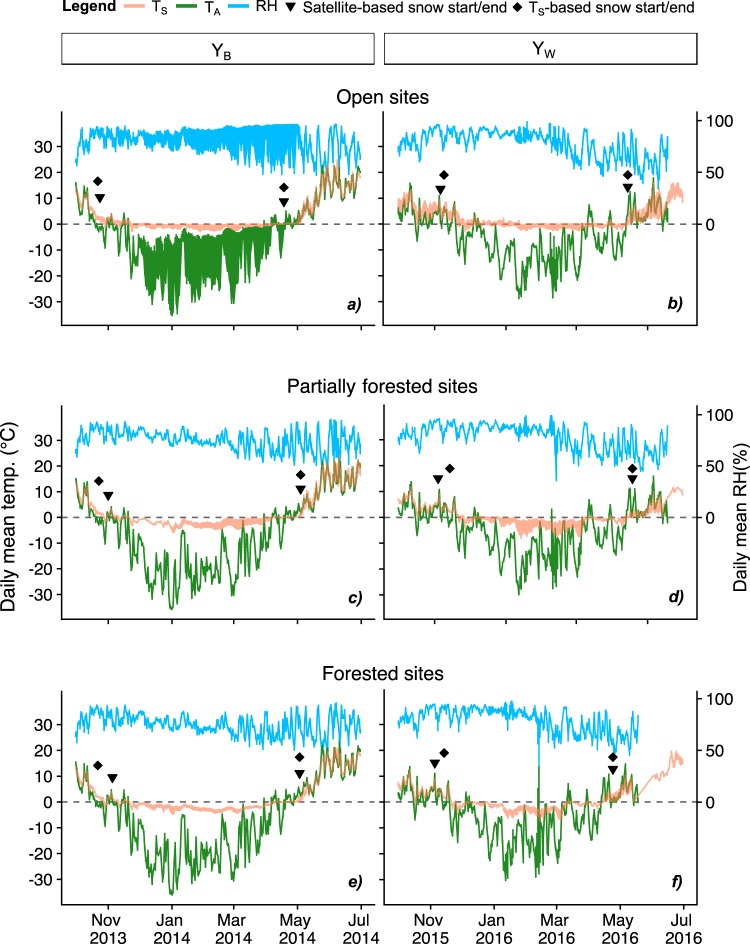
Daily mean T_S_, T_A_, and RH under different site conditions in Y_B_ and Y_W_. Daily mean T_S_ and T_A_/RH values are calculated from hourly data of 5 plots each with 8–9 sensors and one sensor, respectively. (**a**–**f**) show how T_A_/RH is related to T_S_ with respect to snow start/end dates estimated from both satellite (synthetic Landsat images by fusing MODIS and Landsat 8 data) and T_S_ data. For simplicity error estimates of the daily mean values are not shown (see Supplementary Fig. S7 for daily mean T_S_ error estimates).

Field measurements (Fig. 2) and secondary data (Fig. 1) indicate that Y_W_ successfully represented a warm future year in the northern high latitude ecosystems. Consistent with climate model predictions, differences in winter T_A_ between Y_W_ and Y_B_ were particularly large: average winter T_A_ in open, partially forested, and forested sites were 6.58 ^°^C, 9.17 ^°^C, and 9.46 ^°^C higher (p < 0.05), respectively, in Y_W_ than those in Y_B_ (Fig. 2).

**Figure 3:**
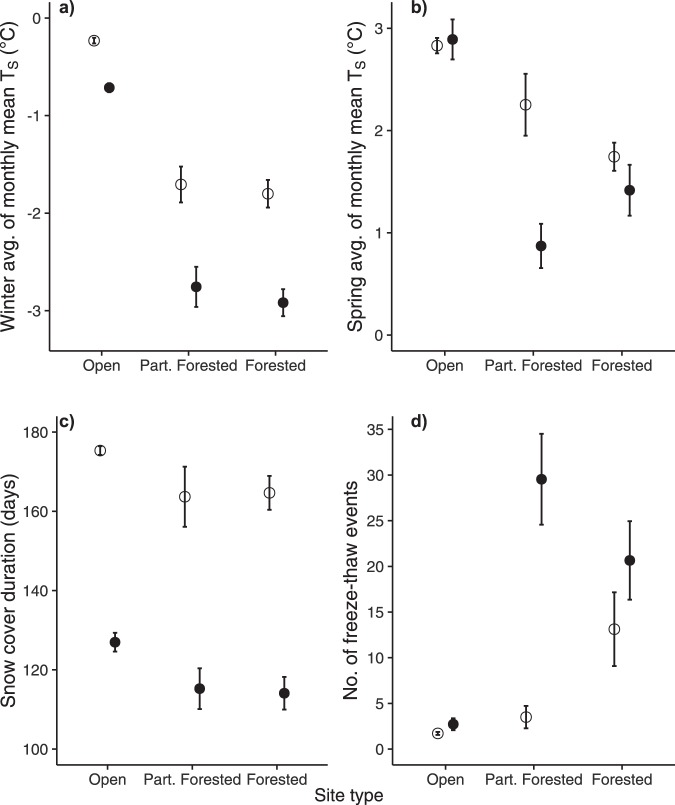
T_S_, snow cover duration, and number of freeze-thaw events in different site conditions in Y_B_ (open circles) and Y_W_ (closed circles). (**a**,**b**), average winter (December–February) and spring (March–May) T_S_, respectively, in Y_B_ and Y_W_. Average summer and fall T_S_ along overall seasonal T_S_ trends for each year are presented in the Supplementary Fig. S4. (**c**), mean snow cover duration for each year estimated from daily T_S_ ranges and maximum values. (**d**), average number of freeze-thaw events for each year. Each data point (with standard error) in (**a**,**b**) is calculated from monthly means over the season, and in (c,d) is calculated from daily T_S_ data of 5 plots each with 8–9 sensors.

Sensor data indicate that average winter T_S_ were significantly lower in Y_W_ compared to Y_B_ (Fig. 3a). In open, partially forested, forested sites, respectively, average T_S_ in Y_W_ was 0.43 ^°^C, 1.22 ^°^C, and 1.13 ^°^C lower (p < 0.01) in Y_W_ than those in Y_B_. Average minimum and maximum winter T_S_ also showed similar patterns under all site conditions (Supplementary Results and Fig. S5a). The differences in average spring T_S_ between Y_B_ and Y_W_ were not as consistent (Fig. 3b). In Y_W_ they were 1.54 ^°^C lower (p < 0.01) in partially forested sites, but marginally higher (0.12 ^°^C, p = 0.38) in open sites, and lower (0.34 ^°^C, p = 0.2) in forested sites than those in Y_B_. In Y_W_ average spring minimum T_S_ were consistently lower and maximum T_S_ showed similar patterns as mean T_S_ compared to Y_B_ (Supplementary Results and Fig. S5b). Overall seasonal patterns in mean and average minimum/maximum T_S_ in different site conditions throughout Y_B_ and Y_W_ are presented in Supplementary Figs S4 and S6, respectively.

Snow cover started earlier and lasted longer in Y_B_ than in Y_W_. T_S_-based snow cover estimates show that in Y_B_ snow started on average 18, 22, and 23 days earlier (p < 0.01) in open, partially forested, and forested sites, respectively, than in Y_W_. Likewise, in Y_B_ snow ended generally on average 3, 16, and 9 days later in open, partially forested, and forested sites, respectively, than in Y_W_. T_S_ and satellite-based estimates of snow start/end dates agreed well with each other with some year- and site-specific variations (Supplementary Table S3). Satellite-based snow start dates for Y_B_ were 2–14 days later than T_S_-based estimates, which were 3, 10, and 9 days earlier in open, partially forested, and forested sites, respectively, for Y_W_. Snow end dates had the least disagreement of only ±1 day for both years across all site conditions (Fig. 2). T_S_-based snow cover duration (SCD_ST_) estimates suggest that SCD_ST_ in Y_B_ were 48, 49, and 51 days longer (p < 0.01) in open, partially forested, and forested sites, respectively, than those in Y_W_ (Fig. 3c). The number of FTEs was substantially higher in Y_W_ compared to Y_B_ (Fig. 3d), increasing by 53% (p = 0.86), 657% (p < 0.01), and 69% (p = 0.07) in open, partially forested, and forested sites, respectively.

## Discussion

Y_W_ average winter T_S_ at 1–2 cm depths were, depending on site conditions, 0.43–1.22 ^°^C lower than those in Y_B_ (Fig. 3a). This result clearly supports the ‘cold soil in a warm world’ hypothesis in the boreal forest context. Snow manipulation studies^2,15,16^ and model results^18,19^ in other ecosystems have also found evidence in support of this hypothesis. Although studies have predicted a rise in spring T_S_^5,17^, our data suggest that Y_W_ average minimum T_S_ was 0.45–1 ^°^C lower compared to Y_B_ with a considerable reduction in magnitude with increasing Leaf Area Index (LAI) (open to forested sites). In contrast, spring mean and maximum T_S_ did not exhibit any consistent pattern (Fig. 3b and Supplementary Fig. S5b). The opposite pattern in spring minimum and maximum T_S_ and the number of days with daily average T_S_ ≤ −5 ^°^C (Fig. 3d), corresponds to a higher frequency of FTEs in Y_W_ than in Y_B_. This effect was more pronounced in forested sites than in open sites. Climate-warming-induced spring FTEs have been suggested by other studies^5,10^. Cold winter soil and frequent FTEs in warm future years are likely to substantially impact terrestrial plants and microorganisms^8^. Winter soil freezing can adversely affect tree growth and functioning^21^ and alter soil carbon dynamics^11,12^. FTEs have also been reported to increase nitrogen mineralization in high-latitude ecosystems^11,12^.

Snow cover and its interaction with forest stand structure were the major drivers of T_S_ differences between Y_W_ and Y_B_ in the present study. Early soil freezing events were associated with delayed snowfall in Y_W_ (Fig. 2). Likewise, reduced SCD_ST_ (by 48–51 days) (Fig. 3c), higher relative humidity (RH) (5.55–9.15%; indicative of high latent heat from melting snow) (Fig. 2), and data from nearby weather stations^22^ (maximum snow thickness and total precipitation in winter and spring were ~40 cm and 190.5 mm, respectively, in Y_W_ and were ~100 cm and 165.3 mm, respectively, in Y_B_) imply that reduced insulation from thinner or less spatially continuous snow cover decreased T_S_^2,6,15^ in Y_W_ compared to Y_B_. It is also evident from Fig. 2 that T_S_ in Y_W_ was tracking T_A_ more closely than in Y_B_^2^. Higher forest cover was associated with an increased magnitude of differences in T_S_ and number of FTEs between Y_W_ and Y_B_ (Figs 2 and 3a,b,d). It is likely that in Y_W_ with a shallower snowpack tree stems and other vegetation cover reduce T_S_ by creating small pockets in the snowpack that allow penetration of cold air into the subnivean space, increasing FTE frequency by a ‘tree well effect’^23,24^.

‘Proxy year’ or ‘analog year’ approaches have been widely used to examine potential effects of climate change on hydrology and agriculture, but only recently applied to ecological processes^25^. This approach allowed us to test the ‘cold soil in a warm world’ hypothesis for the first time in the boreal forest by realistically simulating composite effects of future climate warming^8^. Although we found reduced T_S_ at shallower depth under warming conditions, the findings are still consistent with projected long-term warming in the deep soil. T_S_ at shallower depths are prone to rapid changes modulated by soil moisture and insulating effects of snow, litter, and vegetation^26^, while deep soils respond to the integrated transfer of thermal energy. Because of soil’s high thermal capacity and low heat conductivity, diurnal/seasonal T_S_ changes attenuate with increasing depth and lag considerably behind those of shallower soils. Studies have found the usual soil frost depth is ~15 cm in high-latitude ecosystems^27^. We thus can assume the ‘cold soil in a warm world’ effect is limited to a similar depth. Since carbon in boreal forest soils is primarily stored in the uppermost soil horizons and organic layer^28^, wintertime reductions in surface T_S_ might create a negative climate feedback by reducing soil heterotrophic respiration^6,12^. By assuming simple linear relationships between T_A_ and T_S_, most existing models will miss this feedback and likely over-estimate warming effects on soil C loss. Conversely, increases in FTE are predicted to negatively affect boreal forest regeneration and productivity, which could constitute a positive climate feedback. The insights from our study are thus an important input to development of credible predictions of climate-induced T_S_ change at shallow soil depths that are most important to carbon processes in high-latitude ecosystems^14^.

## Methods

### Study area

Chronosequence plots were established in the boreal forest of northwestern Ontario, Canada ~200 km north of Thunder Bay and 100 km south of Armstrong. Three 10-m radius circular plots (314.15 m^2^) were established in each of two post-fire (fires occurred in 1998 and 2006) and three post-harvest (harvested in 1998, 2006, and 2013) stands (Supplementary Fig. S1); microenvironmental measurements were made from July 2013 until June 2017.

The study area is generally flat with an average elevation of 416 m a.s.l. The soil in this area is a moderately deep Brunisol (coarse loamy texture) with organic layer thickness (LFH) 1–25 cm^29^ and average pH ~5.3. The growing season for this area varies from 110–120 days^29^. Climate normals for annual temperature and precipitation (measured at Armstrong), and snow depth (measured at Thunder Bay) are –1.1 ^°^C, 738.4 mm, and 9 cm, respectively. Mean annual daytime and nighttime windspeeds, measured at Armstrong at 10 m aboveground over the study period, were 0.7 ms^−1^ (maximum 1.2 ms^−1^) and 0.4 ms^−1^ (maximum 1 ms^−1^), respectively^22^.

The study area is a mixedwood boreal forest characterized by trembling aspen (*Populus tremouloides* Michx.), black spruce (*Picea mariana* (Mill.) BSP), white spruce (*P*. *glauca* (Moench) Voss), jack pine (*Pinus banksiana* Lamb.), eastern white cedar (*Thuja occidentalis* L.), balsam fir (*Abies balsaema* (L.) Mill.), and paper birch (*Betula papyrifera* Marsh.). Stand structural attributes are presented in Supplementary Table S1.

### Instrumentation and measurements

Plots were established in locations with at least 1 ha of identical disturbance (either harvest or fire) of similar age-class and were at least 1 km away from each other and from any water body. We used fire maps (obtained from the Ontario Ministry of Natural Resources) and forest management plans (obtained from Resolute Forest Products) to collect information about the forest management history, disturbance type, and stand age in aiding the selection of plot locations.

At the center of each plot a micrometeorological tower was set up to measure air temperature (T_A_) and relative humidity (RH) every hour at 1.5 m height from the ground (data collected using a LogTag HAXO-8; range (T_A_/RH): –40 to +85 °C/0 to 100%; minimum accuracy (T_A_/RH): ±0.5 °C/0.1%). Additionally, we installed nine soil temperature (T_S_) sensors (LogTag TRIX-8) in each plot at ~1–2 cm soil depth (following the guidelines of Lundquist and Lott^24^), which recorded measurements at hourly intervals (Supplementary Fig. S2). The sensors used are rated by the manufacturer from –40 to +85 °C with a minimum accuracy: ±0.5 °C; in lab calibration trials we found the RMSE to be ±0.11 °C in the range –10 to 35 °C (see Supplementary Texts for accuracy reports on this sensor). Each T_S_ sensor was sealed in thin (0.09 mm) waterproof plastic film and was placed at least 50 cm away from tree trunks. Sensor locations were recorded as bearings from the center of the plot and marked with flagging stakes. Microclimatic data were collected annually in summer, and any compromised sensors were replaced.

Leaf Area Index (LAI) was determined using hemispherical photographs (HPs) taken with a Nikon CoolPix 4500 (4 Megapixels) camera with a Nikon FC-E8 fisheye converter (angle of view 183°) mounted on a tripod. Except in 2013, summer and winter HPs were taken each year in early July and late September/October, respectively, in three equally spaced locations within each plot at 1 m above ground as shown in Fig. S2. Exposure settings and analysis of HPs, using Gap Light Analyser^30^, were done as per the guidelines of Zhang *et al*.^31^. The average of the three LAI-4 (LAI estimated over zenith angle 0–60°) values was used as the LAI for a plot in a given season/year.

Stand density was measured every year as the number of trees (diameter at breast height ≥5 cm and height >1 m) within each plot and converted to stems/ha. Heights (m) of these trees were measured every year using a Suunto PM-5 Clinometer. Similarly, litter depths (mm) were measured using a ruler in locations adjacent to each soil temperature sensor within each plot. We set up four 1-m^2^ subplots within each plot and visually determined the percent cover of ground-layer vegetation every year (Supplementary Fig. S2).

### Proxy year establishment

Proxy baseline (Y_B_) and future warm (Y_W_) years were determined by comparing local (study area) T_A_ anomalies with the northern hemisphere (NH) anomalies. The HadCRUT4 NH monthly T_A_ anomaly data^32^ over 1840–2017 were used for this purpose. A simple polynomial regression curve was fitted to the annual NH T_A_ anomaly data to show a general trend over the 21^st^ century (Fig. 1). The GHCN-D (v2) (Global Historical Climatology Network - Daily data) daily average T_A_ data^33^ for weather stations around the study area (48°–50° N and 88°–90^o^ W) were analysed via the KNMI Climate Explorer (https://climexp.knmi.nl) platform to calculate local monthly T_A_ anomalies with respect to the 1961–1990 baseline year (since HadCRUT4 anomaly data are also based on 1961–1990).

Results from a one-way ANOVA with robust estimation indicated that December 2013–November 2014 had the lowest annual T_A_ anomaly among the years over the study period (2013–2017) and did not differ significantly (p = 0.11) from NH average annual anomaly. December 2015–November 2016, however, had the highest T_A_ anomaly for all seasons over the study period and the annual T_A_ anomaly was significantly higher (p < 0.01) than the NH average annual anomaly (Fig. 1). These years are representative of the historical baseline years and projected warm future years. So, for this study, we chose December 2013–November 2014 as the Y_B_ and December 2015–November 2016 as the Y_W_.

### LAI-based site categorization

To assess the generality of the ‘cold soil in a warm world’ hypothesis in different site conditions, we classified plots based on their LAI values as: ‘open’, ‘partially forested’, and ‘forested’. K-means clustering algorithm was used to determine the LAI cluster centers; lowest center value was assigned to ‘open’, medium value to ‘partially forested’, and highest value to ‘forested’. The LAI cluster center for the summer was 0.05 in open, 0.72 in partially forested, and 1.36 in forested sites. The winter LAI cluster center was 0.05 for open, 0.42 for partially forested, and 0.79 for forested sites.

### T_S_-based snow cover duration (SCD_ST_)

To determine snow cover duration (SCD_ST_) from T_S_ for each sensor in each plot, hourly sensor data were converted to daily T_S_ ranges (*ΔT*_*S*_* = daily maximum T*_*S*_ *– daily minimum T*_*S*_). If *ΔT*_*S*_ remained ≤1 ^°^C over 48 hours and the daily maximum T_S_ was <2 ^°^C, we considered ‘snow present’ for that day. The resulting daily snow present/absent time series were checked against T_S_ sensor data and snowfall event data from nearby Armstrong airport weather station to ensure prediction quality. A number of studies^24,34,35^ have successfully used similar algorithms to determine SCD_ST_.

### T_S_, T_A_, RH, SCDST, and freeze-thaw events (FTEs) data analysis

Hourly sensor data were first converted to daily mean, minimum, and maximum values that were then used to calculated plot-wise monthly mean, minimum, and maximum T_S_/T_A_/RH for each sensor. Plot-wise seasonal mean T_S_, average minimum and maximum T_S_, and mean T_A_/RH for each sensor were calculated from monthly data. Seasons in this study were defined as: winter (December–February), spring (March–May), summer (June–August), and fall (September–November).

The frequency of freeze-thaw events (FTEs) for each year in all site conditions were calculated as the number of days with daily average T_S_ ≤ −5 ^°^C (there was no more than 1 FTE per day). Instead of using T_S_ < 0 ^°^C, we choose −5 ^°^C because studies have found that at T_S_ ≤ −5 ^°^C soil microbial activities are inhibited substantially in high-latitude ecosystems^11^.

Site-specific differences in T_S_, T_A_, RH, SCD_ST_, and FTE between Y_B_ and Y_W_ were tested using linear mixed effect (LME) models. For comparison of T_S_ we focused both on mean and minimum/maximum values, because in projected future warm years maxima/minima of the extreme climate events can have more serious consequences for plants and microorganisms than changes in projected mean values^1^. In LME models, sensor replications nested within each plot were considered random effects, and proxy year and site conditions (and their interactions) were considered as main effects. Dependent variables (T_S_, T_A_, RH, SCD_ST_, FTE) were log-transformed where necessary to meet the residual normality assumption of LME models. All analyses were preformed using the R language platform^36^.

### Snow cover duration from satellite data (SCD_S_)

Remote sensing assessments of snow cover duration in spatially heterogeneous sites require high-resolution spatiotemporal satellite data. None of the freely available satellite images meet this resolution requirement; for example, the MODIS (Moderate Resolution Imaging Spectroradiometer) satellite provides daily global data at a low spatial resolution (maximum 250 m) and the Landsat satellites provide high spatial resolution (30 m) global data at a 16-day interval (pixels are often cloud contaminated). Thus, integrating high-temporal-resolution MODIS data with high-spatial-resolution Landsat data to produce synthetic data with high spatiotemporal resolution is necessary to study highly dynamic land surface processes that operate at a small scale.

MODIS-Landsat fusion has been achieved by a number of models and algorithms, including the Spatial and Temporal Adaptive Reflectance Fusion Model (STARFM)^37^, the Enhanced Spatial and Temporal Adaptive Reflectance Fusion Model (ESTARFM)^38^ and spatiotemporal image-fusion models^39^. We have chosen the STARFM, originally proposed by Gao *et al*.^37^ and tested in a Canadian boreal forest, to generate daily snow cover maps to supplement T_S_-based findings. In this algorithm, a first order approximation of the relationship between coarse MODIS (M) data and Landsat (L) reflectance data for a pixel located at (x_i_, y_i_) and acquired on date t_k_ was assumed as:

$$L\,({x}_{i},\,{y}_{i},\,{t}_{k})=M\,({x}_{i},\,{y}_{i},\,{t}_{k})+{{\epsilon }}_{k}$$

Where ∈_k_ represents error in observed MODIS and Landsat reflectance resulting from differing bandwidth and solar geometry^37^. STARFM is one of the most extensively tested fusion techniques that requires only one MODIS-Landsat pair input (but performs better with two pair input) and requires less computational power than alternative approaches.

### Data preparation for fusing

We used the Normalized Difference Snow Index (NDSI) approach to determine SCD_S_. It is a widely used satellite-image-based spectral index usually calculated from reflectance in green and shortwave infrared bands^40^. To properly set the NDSI threshold in forested areas, Normalized Difference Vegetation Index (NDVI), calculated from the reflectance in red and near infrared (NIR) bands, was used as an auxiliary input in the snow-mapping algorithm^41^. So, for this study, we used green, red, near infrared (NIR), and shortwave bands to map snow cover duration.

Radiometrically, atmospherically, and geometrically corrected MODIS (horizontal tile: 12, vertical tile: 4) (MOD09GA V006)^42^ and Landsat 8 (Level 2)^43^ (WRS2 path/row: 25/26, 26/25) surface reflectance products for the study area over October 2013–May 2014 and October 2015–May 2016 were used in this study. MOD09GA daily surface reflectance data in green (band 4: 545–565 nm), red (band 1: 620–670 nm), NIR (band 2: 841–876 nm), and shortwave infrared 2 (SWIR2) (band 6: 1628–1652 nm) bands were in 500-m resolution (total images = number of bands × day = 4 × 440 = 1760). The equivalent Landsat 8 surface reflectance data in green (band 3: 525–600 nm), red (band 4: 630–680 nm), NIR (band 5: 845–885 nm), and SWIR2 (band 6: 1560–1660 nm) bands were in 30-m resolution, and land cloud cover per scene was less than 20% (total images = 4 × 19 = 76).

The Landsat 8 shares similar sensor geometry with MODIS and both visit the same place at almost the same time. It can thus be assumed that they have an almost identical viewing and illumination geometry, and can be used in the fusion process without further angular adjustments^44^. MODIS images, however, were re-projected to UTM (Universal Transverse Mercator, Zone 16 N) and pixels were resampled (using the nearest neighborhood method) to 30-m resolution to match with Landsat 8 images. MODIS and Landsat 8 images were also precisely co-registered using the common point comparison method and brought into the same spatial extent.

Only cloud and water-body free high-quality pixels were used as input to STARFM. The MOD09GA surface reflectance 500 m quality assurance band was used to mask pixels with a status bit flag other than ‘0000’ for each of the four bands. Similarly, for Landsat 8 images, the level-2 pixel-quality band and radiometric saturation QA bands were used to mask radiometrically saturated, cloud-contaminated, and low-quality pixels. Finally, the pixels with missing values were set to –9999 and images were converted to signed 16-bit binary format. A series of R scripts^36^ was used to prepare satellite images for input in STARFM. C codes to implement STARFM were adapted from Gao *et al*.^37^. Algorithm details and information on data preparation can also be found in Gao *et al*.^37^ and Zhu *et al*.^38^.

### Inputs to STARFM for producing synthetic Landsat 8 images

Two pairs of same-day MODIS-Landsat 8 images within two months either side of the prediction date^45^, along with MODIS image of the prediction date, were used as inputs to STARFM to predict synthetic Landsat 8 images for the dates for which Landsat 8 images were either not available or cloud contaminated (>20%) (Supplementary Fig. S3). Landsat 8 and MODIS equivalent bands were used to produce synthetic Landsat 8 images of the equivalent band. For example, Landsat 8 green (band 3) and MODIS green (band 4) bands were used to produce the synthetic Landsat 8 green-band image.

### Accuracy assessment of predicted Landsat 8 images

To assess the accuracy of STARFM predicted images, synthetic Landsat 8 images were produced in green, red, NIR, and SWIR2 bands for three dates (2013-12-07, 2014-02-16, and 2016-03-18) spanning the study period. Predicted synthetic images were compared pixel-to-pixel with actual Landsat 8 images of the corresponding dates, and Spearman Rho (using the complete observation method), root-mean-square-error (RMSE) and mean absolute error (MAE) estimates were calculated to assess the accuracy. STARFM prediction maintained a reasonable accuracy over the study period compared to other studies^37,45^ (see Supplementary Table S2).

### NDSI-based snow-mapping algorithm

Using the daily high-resolution synthetic data, NDSI was calculated as:

$$NDSI=\frac{reflectance\,in\,green\,band-reflectance\,in\,SWIR2\,band}{reflectance\,in\,green\,band+reflectance\,in\,SWIR2\,band}$$

Similarly, NDVI was calculated as:

$$NDVI=\frac{reflectance\,in\,NIR\,band-reflectance\,in\,red\,band}{reflectance\,in\,NIR\,band+reflectance\,in\,red\,band}$$

Using reflectance property of clouds in SWIR2 band, NDSI can successfully separate clouds from snow. For mapping snow cover with NDSI, a physically based threshold value > 0.4 is usually used to indicate snow cover^40^. There is, however, evidence^46^ that in conifer-dominated forests NDSI < 0.4 can also be snow. Moreover, Hall *et al*.^41^ found that NDSI values < 0.4 can also indicate snow if NDVI value is ~0.1. It is, therefore, important to identify area-specific NDSI threshold values to delineate snow cover.

After extensive visual inspections, using Google Maps, Sentinel-2 images (red, green, and blue bands), and predicted NDVI maps, for this study 1 ≥ NDSI > 0.35 was used for October 2013–May 2014 and 1 ≥ NDSI > 0.3 was used for October 2015–May 2016 to define snow cover. This year-specific NDSI threshold mainly stemmed from snow patchiness as a result of high difference in winter T_A_ and T_S_ between study years^47,48^. To prevent NDSI overestimation, green band reflectance values ≤ 0.1 were masked before NDSI calculation^49^. To prevent snow-cover underestimation, at 0.1 < NDSI < 0.3 it was also considered as snow, only if 0.08 ≤ NDVI ≤ 0.12^41^.

To determine snow-start and snow-end dates, and to compare the plot-wise results with SCD_ST_, decisions on presence/absence of snow were made based on the information (NDSI and NDVI) extracted from each plot centers with a 15-m buffer around it. The NDSI-based snow cover mapping algorithm works only for pixels with at least 50% snow cover^41^. To ensure consistent comparisons between SCD_ST_ and SCD_S_, for SCD_ST_ we consider snow present only if ≥50% of the T_S_ sensors in a plot agreed that there was snow on the particular day.

### Accuracy assessment of NDSI-based snow cover maps

A confusion matrix was generated to show the overall agreement in snow cover duration estimated from T_S_ (SCD_ST_) and satellite (SCD_S_) data (Supplementary Table S3). Considering SCD_ST_ as the reference and SCD_S_ as the predicted, results from the confusion matrix show that 72.8% of the time SCD_ST_ agreed with SCD_S_. The all-weather overall accuracy of our fused daily snow-cover map is higher than the MODIS daily snow products (MYD10A1 and MOD10A1) (31–45%)^50^. It is interesting to note that the overall accuracy of NDSI-based snow cover maps varied between years with a higher overall accuracy in Y_B_ (80%) than in Y_W_ (67%). This suggests that, in a warmer world, spatial variability in T_S_ and snowpack will likely be higher than what it is now^17^, and we may need high-spatiotemporal-resolution satellite images complemented by field measurements^35^ to accurately capture this variability.

### Code availability

Data preparation and all statistical analyses were implemented in the R programming language (version 3.4.3) (ref.^36^). R codes can be requested to M.A.H. (abdul.halim@mail.utoronto.ca).

## Electronic supplementary material

Supplementary Information

## Data Availability

The northern hemisphere HadCRUT4 data used in this study are available from https://crudata.uea.ac.uk/cru/data/temperature/. Landsat 8 (Level 2) and MOD09GA (V006) data can be downloaded from https://earthexplorer.usgs.gov and https://search.earthdata.nasa.gov, respectively. Other data and materials can be requested to the corresponding author.
