# Supplementary material for: A proxy-year analysis shows reduced soil temperatures with climate warming in boreal forest
Source: Sci Rep. 2018 Nov 15;8:16859. doi: 10.1038/s41598-018-35213-w (PMC6237965; doi:10.1038/s41598-018-35213-w)
Supplement: Supplementary file 1 — Supplementary Information [file 41598_2018_35213_MOESM1_ESM.pdf]

Supplementary Information

A proxy-year analysis shows reduced soil temperatures with climate warming in boreal forest

Md Abdul Halim[1,2*] and Sean C Thomas[1]

[1]University of Toronto, Faculty of Forestry, 33 Willcocks Street, Toronto, ON M5S 3B3, Canada.

[2]Shahjalal University of Science and Technology, Dept. of Forestry and Environmental Science, Sylhet-3114, Bangladesh.

* Corresponding author: abdul.halim@mail.utoronto.ca

SUPPLEMENTARY RESULTS

Supplementary Fig. S4 provides overall seasonal T_S trend throughout the year in Y_B and Y_W under different site conditions. It indicates that summer (June–August) and Fall (September–November) mean T_S were 1.2–1.63°C (partially forested, open, forested sites) and 1.95–2.62°C higher ($p < 0.01$), respectively, in Y_W than those in Y_B. Winter and spring mean T_S are presented in more details in the main text (Fig. 3a–b).

Supplementary Fig. S5 depicts winter (December–February) and spring (March–May) average minimum and maximum T_S in different site conditions. Supplementary Fig. S5a shows that average minimum and maximum winter T_S in Y_W were 0.62–1.44°C (open to forested sites) and

0.34–1.18°C (open ($p = 0.11$), forested, and partially-forested sites) lower ($p < 0.01$), respectively, than those in Y_B. Supplementary Fig. S5b shows that average minimum spring T_S in Y_W were 0.45–1°C (forested ($p = 0.07$) to open sites) lower ($p < 0.01$) than those in Y_B. Average maximum spring T_S in Y_W were 1.17°C higher ($p < 0.01$) in open sites, 2.52°C lower ($p < 0.01$) in partially forested sites, and indistinguishable (0.04°C lower, $p = 0.93$) in forested sites, compared to Y_B.

Complementary to the Supplementary Fig. S5, the Supplementary Fig. S6 shows overall seasonal trends in average minimum and maximum T_S in different site conditions. Summer and fall average minimum T_S were 2.37–2.97°C (open to forested sites) and 2.24–2.34°C (partially-forested, open, forested sites) higher ($p < 0.01$), respectively, in Y_W than those in Y_B. Average maximum summer T_S did not show any consistent pattern: in Y_W they were 0.17°C lower ($p = 0.7$) in open sites, 0.37°C higher ($p = 0.52$) in partially forested sites, and 0.12°C higher ($p = 0.86$) in forested sites those in Y_B. Average maximum fall T_S were however 1.56–3.28°C (partially-forested, open, forested sites) higher ($p < 0.01$) in Y_W compared to Y_B.

SUPPLEMENTARY TEXTS

Accuracy assessment of LogTag TRIX-8 sensors

To assess the measurement accuracy of the LogTag TRIX-8 sensors (LogTag, Auckland, New Zealand) (reported minimum accuracy: ± 0.5°C over −20 to 40°C; resolution: 0.03°C), we have compared them with the high-accuracy factory-calibrated TSYS01-1 digital temperature sensors (TE Connectivity, Schaffhausen, Switzerland) (reported accuracy: ± 0.1°C over –20 to 70°C; resolution: 0.01°C). TSYS01-1 sensors have built-in 24-bit ADCs (analog to digital converters) and were interfaced with ATMEGA328p microcontrollers to read the sensor ADC values and calculate temperatures based on supplied calibration parameters. Eight (i.e., the same number used in the study plots) LogTag TRIX-8 sensors and three TSYS01-1 sensors sealed in 0.09 mm waterproof plastic film (identical to that used in the study plots) were placed in a Fisher Serological Bath containing a 25% salt (NaCl) solution in deionized water. Ice cubes were added to the salt water to reduce the temperature to –10°C followed by hot water to increase the temperature up to 35 °C. The mixture was intermittently stirred; sensors were programmed to record measurements synchronously every minute.

Results from a simple linear regression indicated that LogTag TRIX-8 and TSYS01-1 corresponded exceptionally well ($R^2 = 0.999$, RSME = 0.117) with intercept of –0.053 ($p < 0.01$) and slope of 0.998 ($p < 0.01$) (Supplementary Fig. S8). Although LogTag TRIX-8 were found highly accurate, the significant intercept term indicates a slight negative bias. Under similar thermal conditions, both LogTag TRIX-8 (standard errors ranged from 0.020 to 0.097°C) and TSYS01-1 (standard errors ranged from 0.000 to 0.096°C) sensors had high agreement within themselves. Given the high accuracy and resolution of the LogTag TRIX-8 sensors over the –10

to 35°C range (with 8–9 replicates for each plot), we can safely consider two significant digits after decimal for temperature values.

SUPPLEMENTARY FIGURES

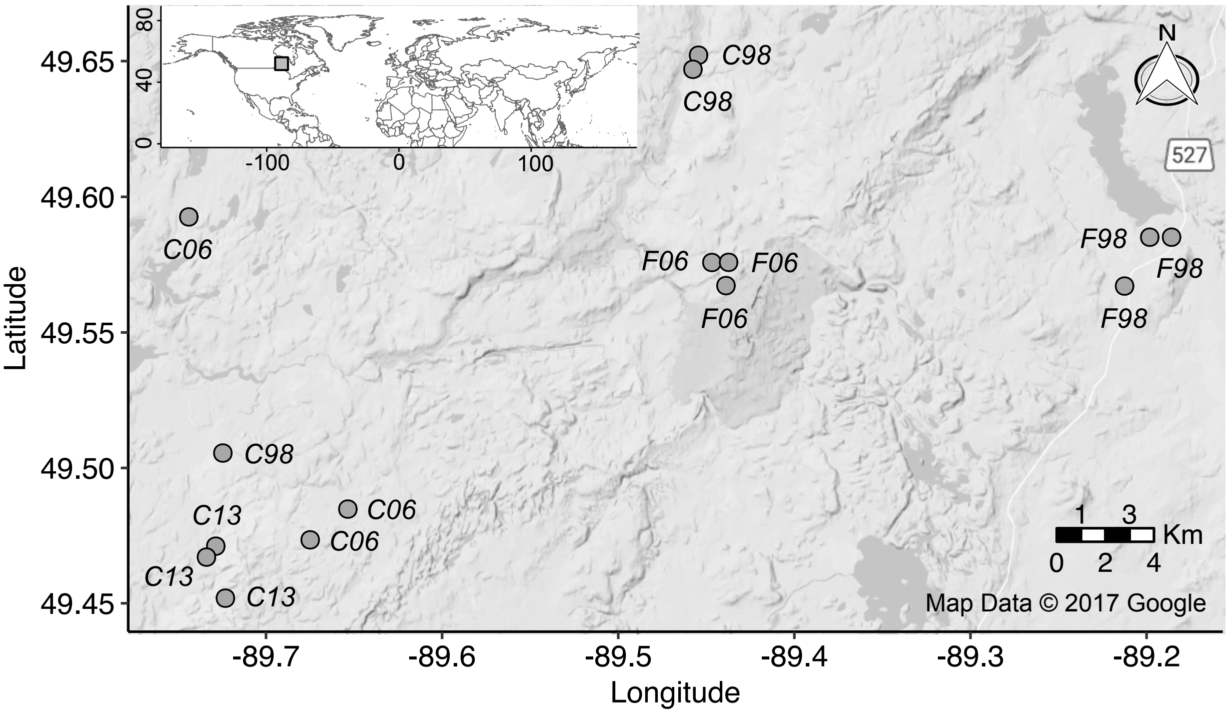

Supplementary Figure S1. Map of the study area showing plots with their originating disturbance types and years. Disturbance type C: Clearcut, F: Fire, and year 98: 1998, 06: 2006, and 13: 2013. Inset: the square indicating location of the study area on the northern hemisphere map. The scale (not shown) of this map is different than the background map.

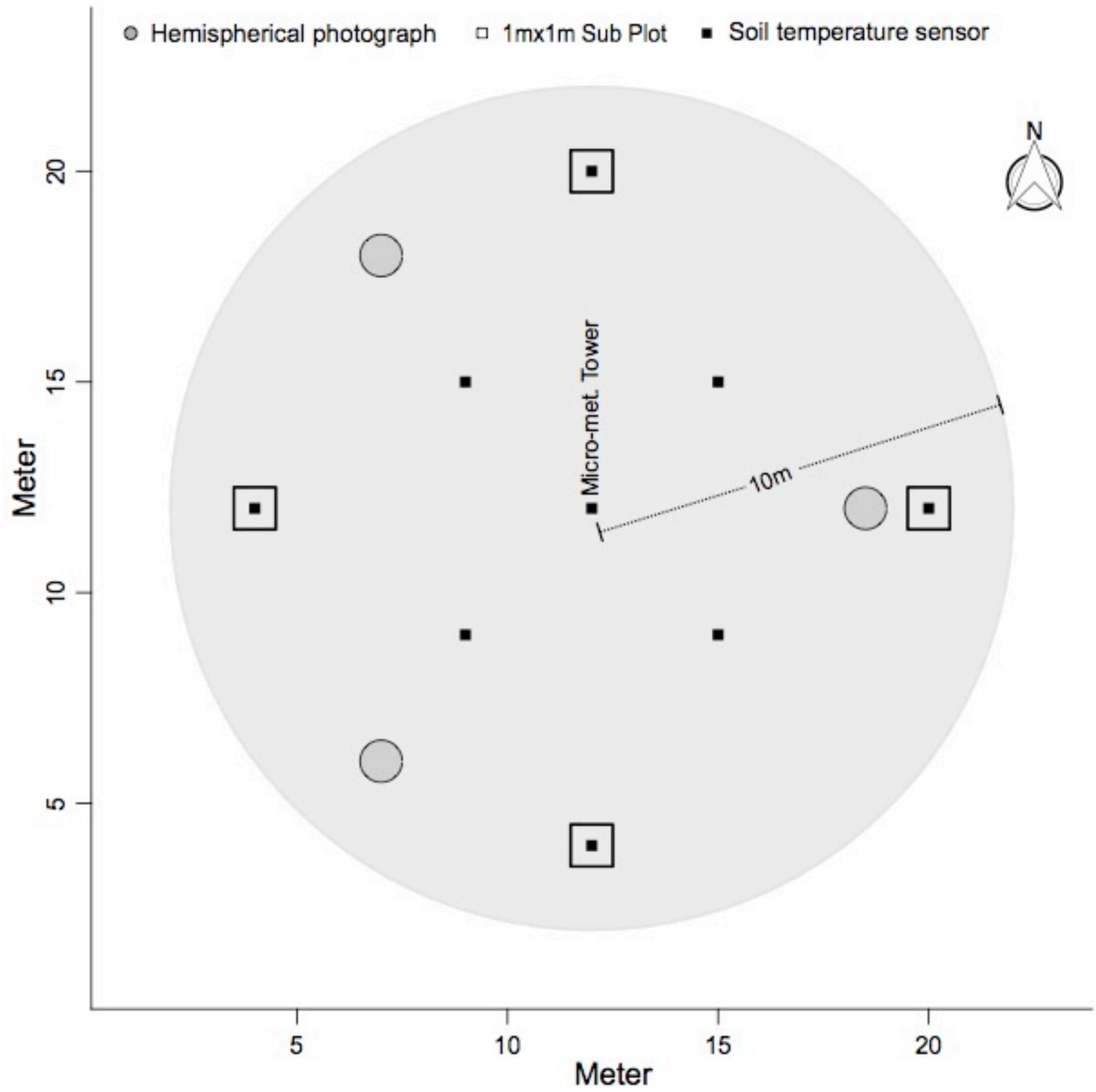

Supplementary Figure S2. Schematic of instrumentation and measurements within each plot in the study area.

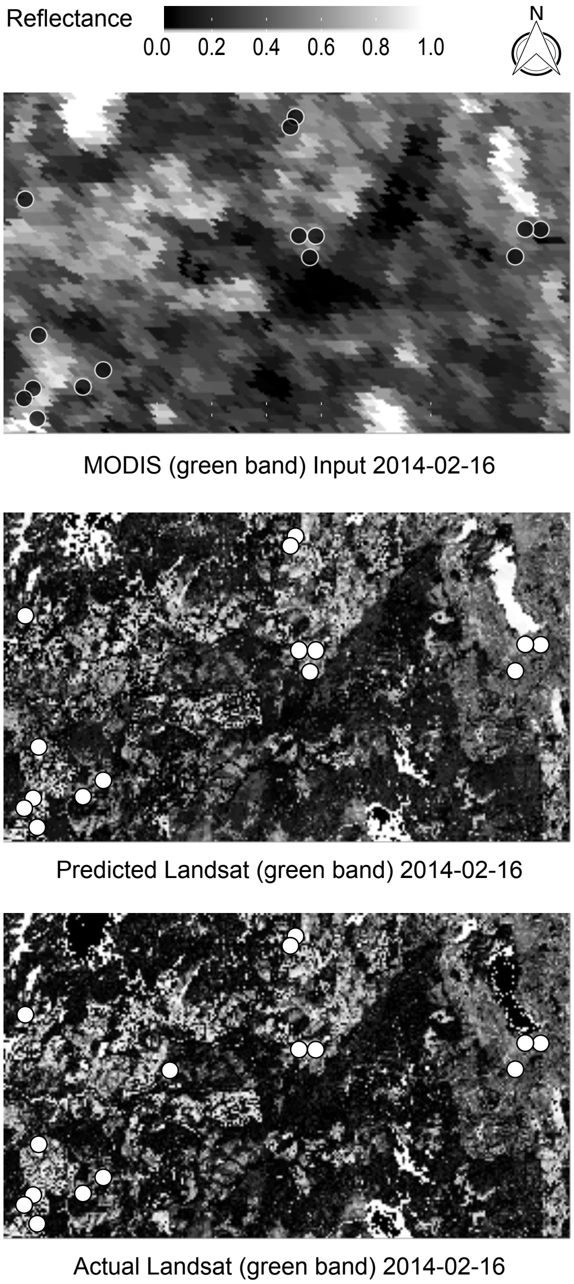

Supplementary Figure S3. STARFM inputs and predicted Landsat 8 surface reflectance. Top: MODIS (500 m) surface reflectance input of the prediction day (2014-02-16). Middle: STARFM predicted Landsat 8 surface reflectance for 2014-02-16 from MODIS-Landsat 8 pair of 2014-01-08 and 2014-03-29 (not shown). Bottom: actual Landsat 8 surface reflectance of the

prediction day. Black/white circles on the images are plot locations. All images are displayed in an identical linear stretch.

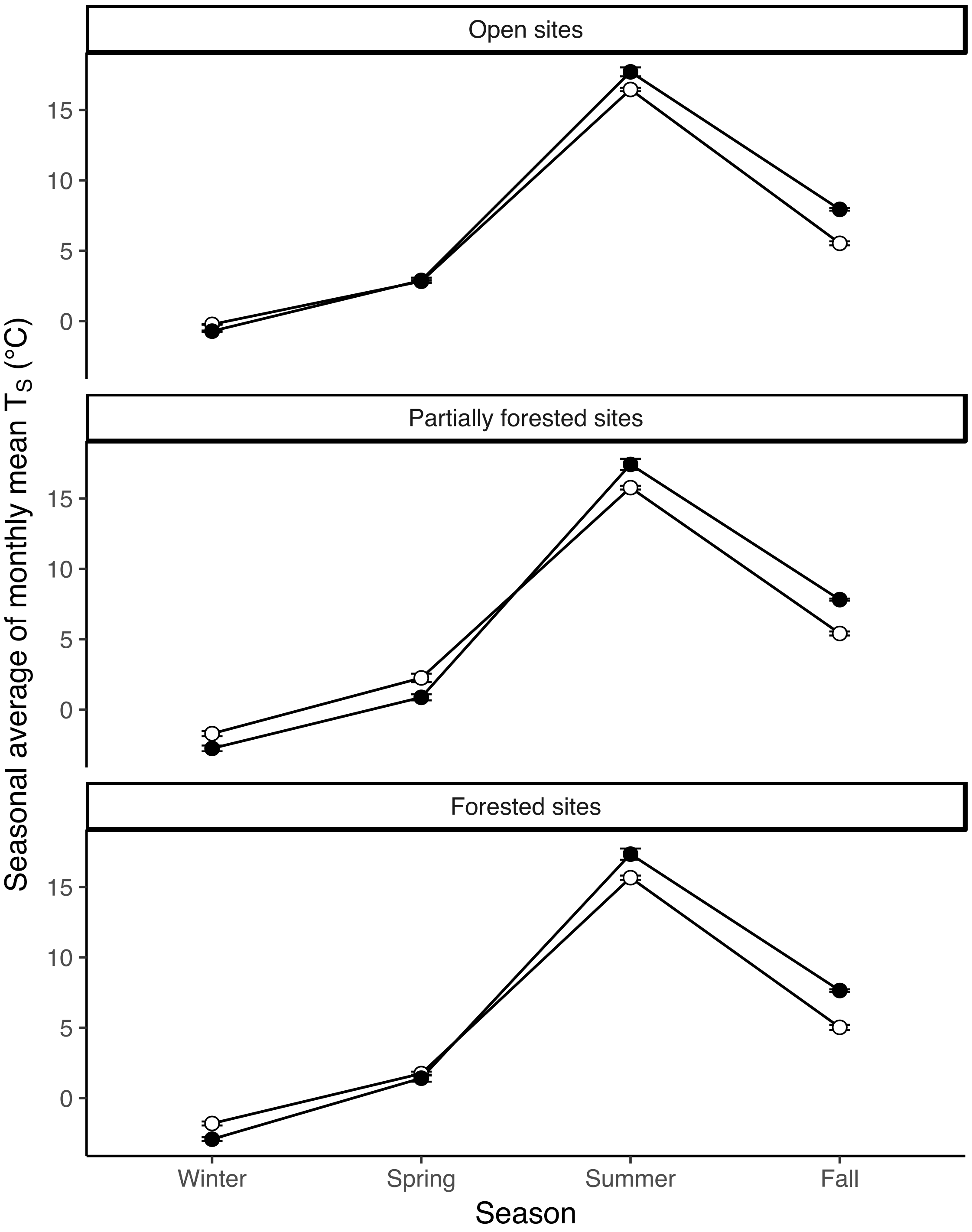

Supplementary Figure S4. Seasonal average T_S trends in Y_B and Y_W under different site conditions. Each data point in the graph is the seasonal average (with standard error) of monthly data from 5 plots each with 8–9 sensors for Y_B (open circle) and Y_W (close circle).

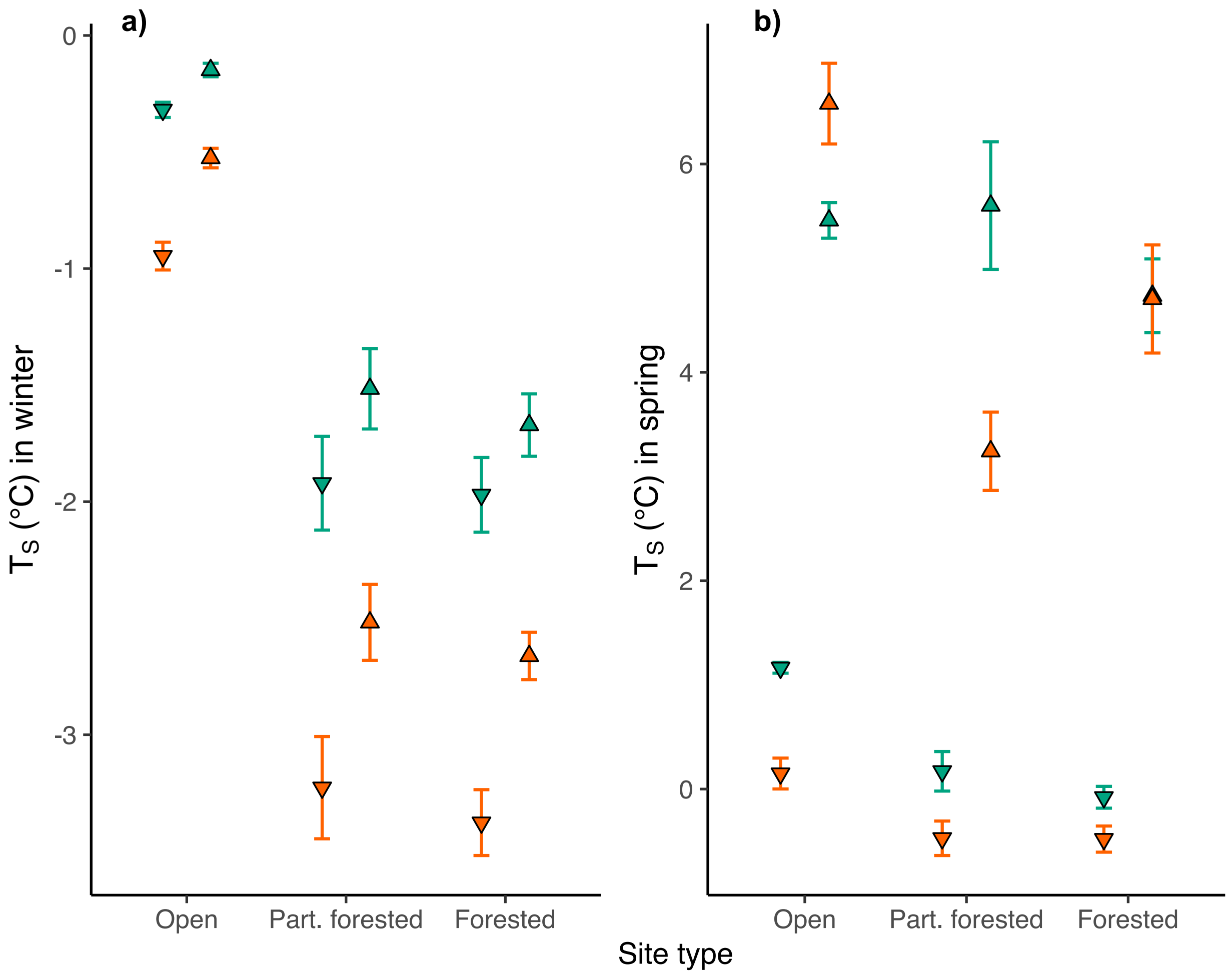

Supplementary Figure S5. Winter and spring average minimum (upside-down triangle) and maximum (right-side-up triangle) T_S in Y_B (green) and Y_W (orange). Each data point in the graph is the seasonal average (with standard error) calculated from monthly means of 5 plots each with 8–9 sensors.

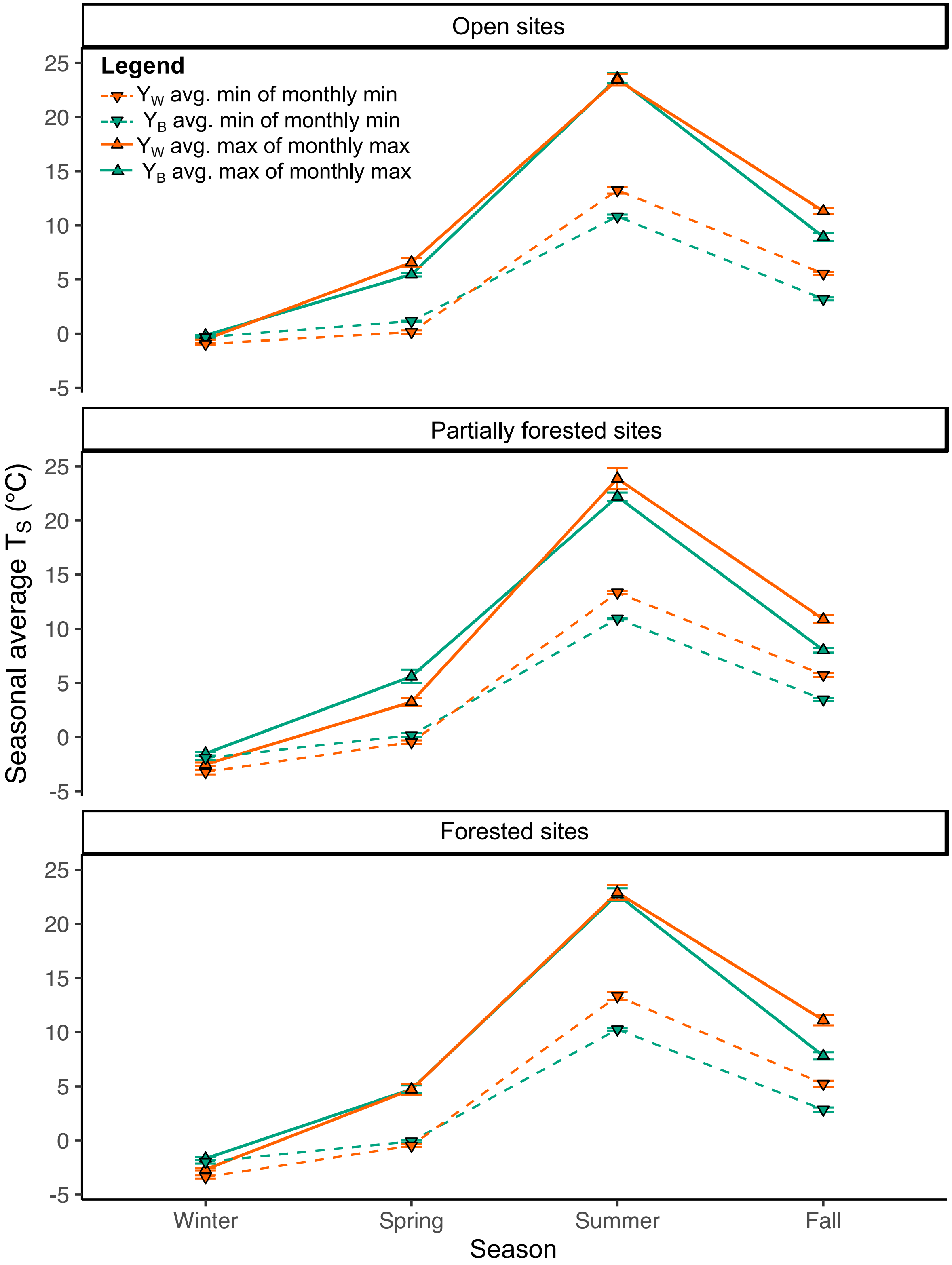

147

148 **Supplementary Figure S6. Trends in seasonal average of monthly minimum and maximum**

T_S of Y_B and Y_W in different site conditions. Each data point in the graph is the seasonal average (with standard error) of monthly T_S from 5 plots each with 8–9 sensors.

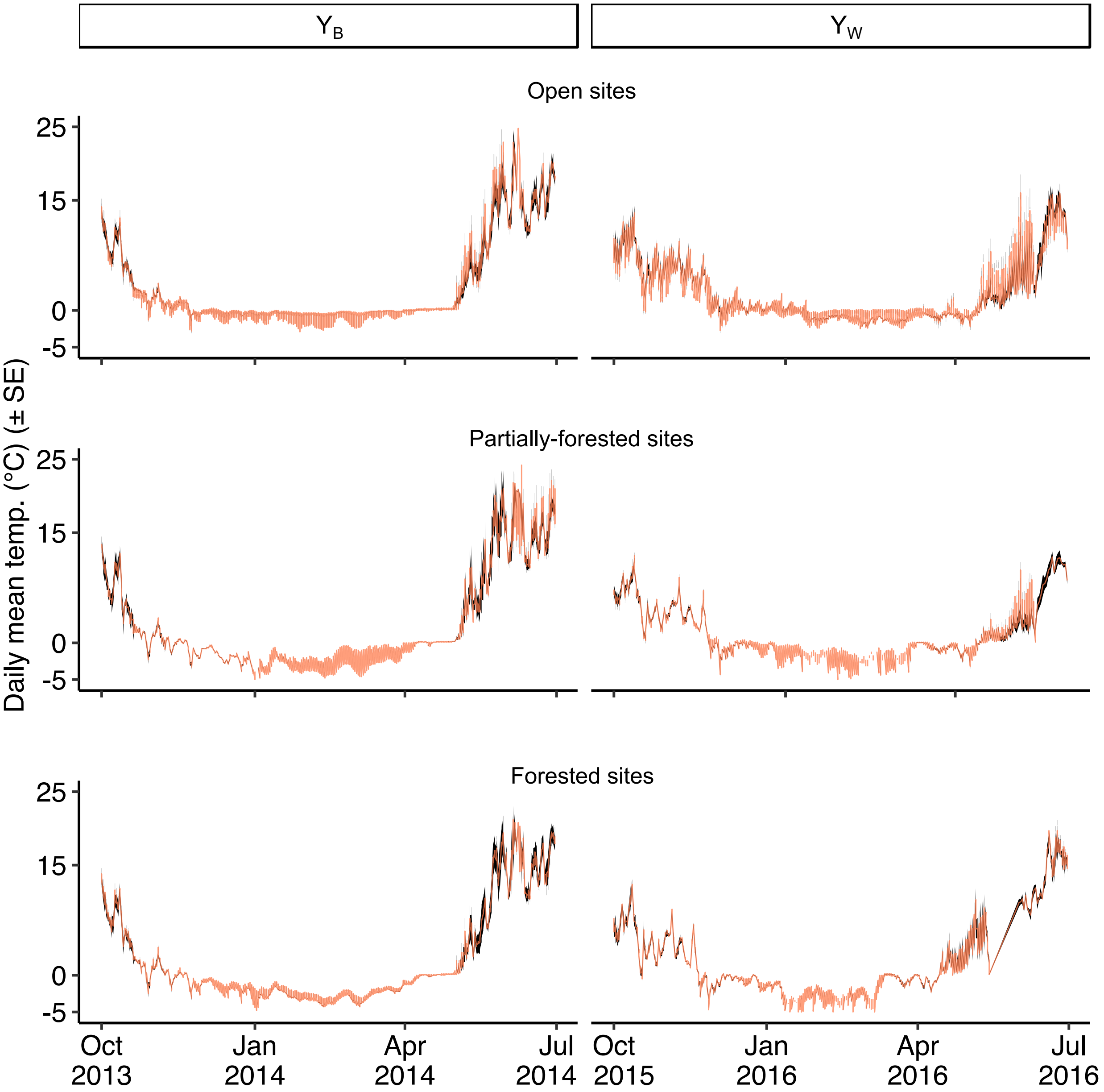

Supplementary Figure S7. Trends in daily mean T_S (°C) in different site conditions in Y_B and Y_W. Brown lines indicate daily mean T_S calculated from hourly data of 5 plots each with 8–9 sensors, and black lines indicate their corresponding standard errors (SE).

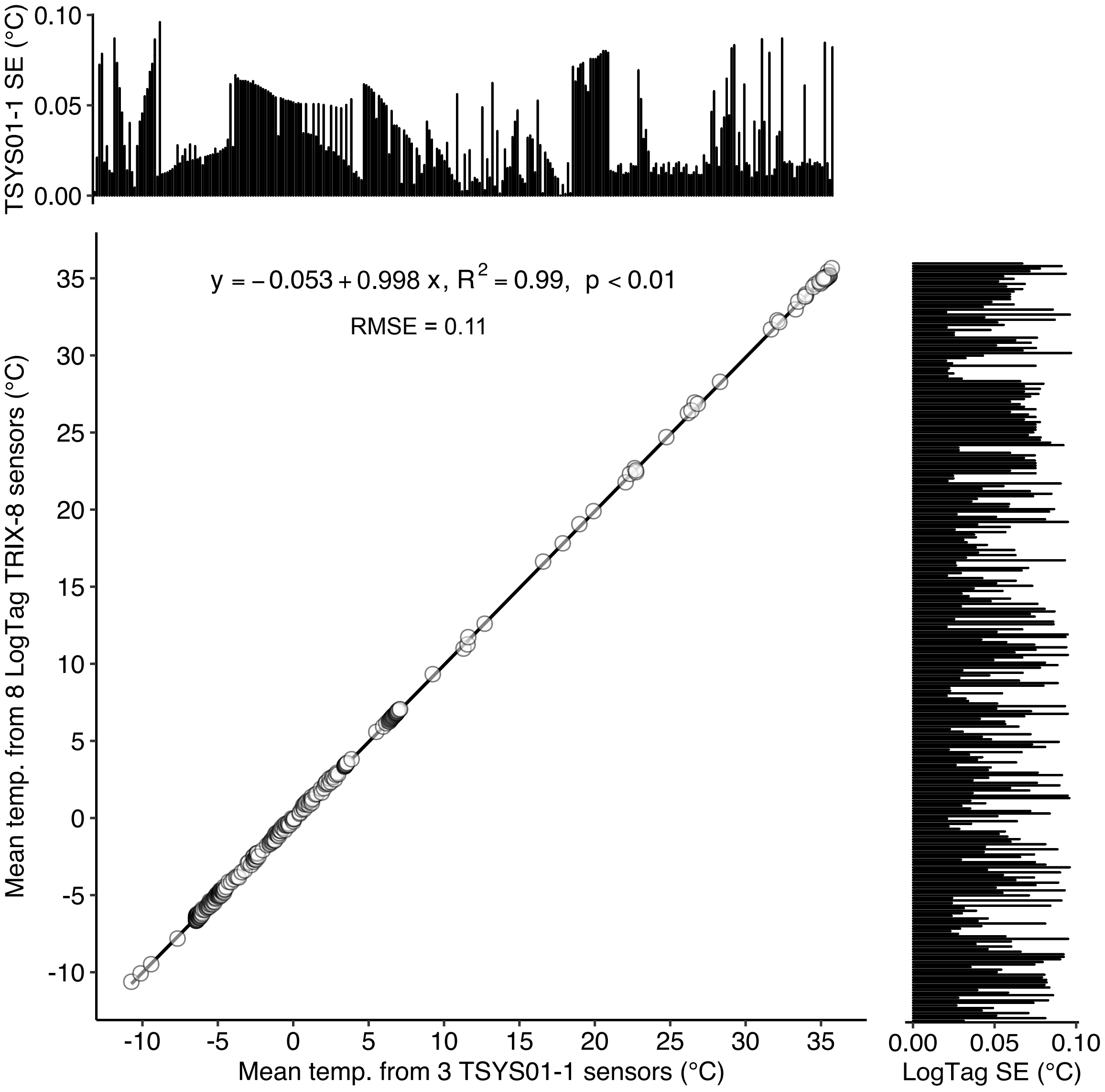

Supplementary Figure S8. Accuracy of LogTag TRIX-8 compared to TSYS01-1 sensors in a salt (NaCl) water bath over temperature range of –10 to 35°C. Marginal graphs (top and right) indicate standard error of each corresponding data point on the scatter plot.

SUPPLEMENTARY TABLES

Supplementary Table S1. Average stand attributes of the study area in Y_B and Y_W under different site conditions. Values in parentheses are standard deviations of mean.

**Stand attributes**	**Open sites**		**Partially forested sites**		**Forested sites**	
	**Y_B**	**Y_W**	**Y_B**	**Y_W**	**Y_B**	**Y_W**
Height (m)	-	-	2.30 (0.85)	3.09 (0.50)	2.37 (0.79)	3.20 (0.50)
Density (stem/ha)	-	-	6733 (3361)	6905 (3246)	7345 (4111)	7540 (4016)
Ground cover vegetation (%)	4.5 (1.4)	34.3 (28.5)	56.5 (16.3)	62.3 (13.0)	66.8 (15.1)	70.9 (14.2)
Litter Depth (mm)	0.80 (0.51)	1.83 (2.12)	3.21 (1.46)	3.76 (1.78)	2.33 (0.78)	2.61 (0.91)

Supplementary Table S2. Average band specific error estimates for the STARFM produced Landsat 8 synthetic images. Values in parentheses are standard deviations of means.

Estimate	Green band	Red band	NIR band	SWIR2 band
Rho[1]	0.839 (0.067)	0.715 (0.100)	0.718 (0.110)	0.721 (0.100)
RMSE[2]	0.098 (0.001)	0.025 (0.006)	0.028 (0.005)	0.024 (0.005)
MAE[3]	0.073 (0.008)	0.020 (0.006)	0.022 (0.005)	0.019 (0.005)

[1]Spearman Rho [2]Root Mean Square Error [3]Mean Absolute Error

Supplementary Table S3. Confusion matrix of the predicted SCD_S with respect to SCD_{ST} as the reference.

Year	Satellite-based snow	T_S-based snow		Overall Accuracy
		Absent	Present	
October 2013–May 2014 to October 2015–May 2016	Absent	337	728	72.8%
	Present	335	2514	
October 2013–May 2014	Absent	92	311	79.8%
	Present	51	1335	
October 2015–May 2016	Absent	245	417	67.0%
	Present	284	1179	
